# Removal of abamectin and conventional pollutants in vertical flow constructed wetlands with Fe-modified biochar†

**DOI:** 10.1039/d0ra08265a

**Published:** 2020-12-15

**Authors:** Nai-Qing Sha, Guo-Hao Wang, Yan-Hong Li, Shao-Yuan Bai

**Affiliations:** Guangxi Key Laboratory of Environmental Pollution Control Theory and Technology, Guilin University of Technology Guilin 541004 China 1020180249@glut.edu.cn; Collaborative Innovation Center for Water Pollution Control and Water Safety in Karst Area, Guilin University of Technology Guilin 541004 China

## Abstract

To improve the ability of constructed wetlands to remove abamectin (ABM) and nutrients, the influence of four different substrates on constructed wetlands was studied. Four vertical up-flow constructed wetlands (UVCWs) were established to treat simulated agricultural wastewater: CW1 (quartz sand + pebbles), CW2 (pebbles + coke), CW3 (Fe-modified biochar + pebbles + coke), and CW4 (unmodified biochar + pebbles + coke). Under different combinations of hydraulic loading and organic loading, CW3 was extremely effective at removing nitrogen compared with CW1, CW2 and CW4. We found that CW3 was the most effective at treating ABM and conventional pollutants. The highest efficiency of removal of abamectin (99%), COD (98%), NH_4_^+^–N (65%), and TP (80%) was obtained in CW3. These results were directly verified by microbiological tests and microbial community analysis. The microbial diversity of CW3 and CW4 was significantly higher than those of CW1 and CW2. Fe-modified biochar provides a feasible and effective amendment for constructed wetlands to improve the nitrogen removal for C/N (2.5 : 1–5 : 1) wastewater by the ability of microbes to remove nitrogen. Fe-modified bamboo charcoal can be used in engineering as a new type of green environmental protection constructed wetland filler in the future.

## Abstract 2

To improve the ability of constructed wetlands to remove abamectin (ABM) and nutrients, the influence of four different substrates on constructed wetlands was studied.

## Graphical abstract

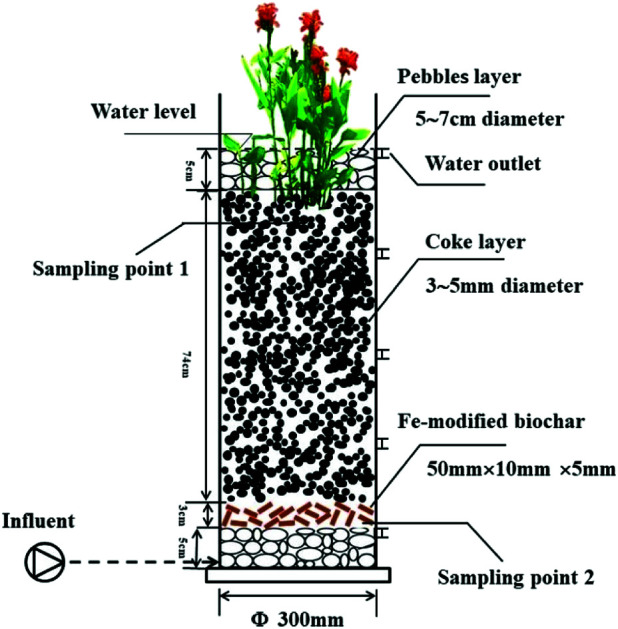

## 1. Introduction

Currently, pharmaceutical compounds have penetrated every aspect of our lives. The abuse of pharmaceutical compounds has had a negative impact on the environment.^1^ The detection and methods for the control of conventional pollutants have matured. Researchers have begun to pay attention to the harm and treatment of emerging pollutants (EOCs) in the environment.^2,3^ The strong ability and persistence of personal-care products (PPCPs, such as antibiotics and sex hormones) and pesticides in the environment have become increasingly prominent.^4,5^ Because of the low concentration of EOCs in the water (μg L^−1^ or ng L^−1^), most water treatment facilities cannot effectively eliminate them.^6^ EOCs and PPCPs and other emerging pollutants have also become new international research hotspots.^7,8^ When pesticides are used in crops, they can reach the soil through rain and river water and then enter the groundwater from the soil through osmosis.^9,10^ This is especially true for antibiotics, since their indiscriminate use has led to the omnipresence of drug-resistant microorganisms and antibiotic resistance genes (ARGs).^11,12^ This situation has received wide attention owing to the potential threat to aquatic ecosystems and human health.^13^ For this reason, the occurrence of PPCPs and pesticides in the aquatic environment has become a worldwide issue of increasing environmental concern.^14^

In nature, organic micropollutants (OMPs) have increasingly emerged in the environment.^15^ For example, pharmaceutical residues and endocrine disrupting compounds are subjected to a series of complex degradative processes, but some of them remain in soil and water owing to their stability.^9^ Some studies have reported the detection of concentrations of OMPs on the order of thousands of nanograms per liter in groundwater.^16^

The pesticide abamectin (ABM, 99% B1a, CAS No. 71751-41-2) is often used to prevent and control agricultural pests.^9,17^ The half-life of ABM in the soil is usually 20–40 d, but depending on the intensity of light, soil type, temperature, and other factors, this parameter will vary.^17^ ABM and its derivatives can be detected in all types of soil environments, including soil, feces, and sediment.^18^ The long-term use of ABM can also cause agricultural non-point source pollution and harm to the aquatic environment.^19,20^ Moreover, these pesticides will form mixtures and byproducts in the environment, causing greater difficulty in the evaluation and treatment of these substances.^21,22^ Therefore, management practices that reduce the potential risks associated with the ecotoxicity of these highly toxic compounds and the demand for cost-effective methods to remove ABM from pesticide wastewater are increasing.^18^ In this study, ABM was selected as a typical micropollutant to investigate the performance of four CWs in the removal of OMPs from wastewaters.

For several types of sewage, such as wastewater, domestic sewage, and agro-industrial waste, constructed wetlands (CWs) are an alternative low-cost new composite treatment technology.^21^ The mechanism of constructed wetlands for removing pollutants usually consist of matrix adsorption, plant absorption, and biodegradation.^8^ As a simple and low-cost technology, CW is often combined with other technologies, such as their integration of septic tanks,^23^ combining up-flow anaerobic sludge technology and CWs,^24^ among others. Most research focuses on plant absorption and microbial degradation, while ignoring the adsorption and removal of substrates. Biochar is the solid product of biomass subjected to pyrolysis, which is often used as an adsorption material for its advantages of high specific surface areas and large pore volumes, and it has recently been approved as a potential carrier of microbial agents.^25,26^ In constructed wetlands, biochar is also often used as a matrix material to improve the efficiency of removal of pollutants.^27,28^ In addition, modified biochar has more activation sites, which increase the electrostatic adsorption of NO_3_–N and enhance its denitrification ability.^4,29^ Iron is an abundant redox-active metal in the earth.^30^ In recent years, researchers have discovered that bacteria play an important role in the anaerobic oxidation of Fe(ii) and can oxidize it to Fe(iii) in an oxygen-deficient environment.^31,32^ Fe-modified biochar is used as a filler in constructed wetlands.

We found that Fe-modified bamboo charcoal adsorbs compounds more effectively because it not only has a large specific surface area and a variety of pores, but it also supports active functional groups of iron oxide on its surface that increase its ability to adsorb compounds. When organic matter is combined with Fe(ii), microorganisms can oxidize Fe(ii) to Fe(iii) to promote the degradation of organic matter.^33^ The Fe-modified biochar has a high affinity for anions and enhances the ability of biochar to remove nitrogen.^29^ The objectives of the study were as follows: (1) to compare the effects of substrate on the performance of CWs in treating different C/N tailwater under various conditions; (2) to evaluate the ability of UVCWs with different fillers to treat ABM; and (3) to compare the microbial community structure of four UVCWs to analyze whether the Fe-modified biochar had an impact on the microbial degradation of ABM.

## 2. Materials and methods

### 2.1. Chemicals

The ABM (99% purity) used in this experiment was purchased from Yeyuan Reagent (Shanghai, China). The reagents used to detect ABM were all high-performance liquid chromatography (HPLC) grades. Ethanol, ferric nitrate, and the other chemicals were of analytical grade.

### 2.2. Preparation and characterization of Fe-modified biochar

First, bamboo was cut into small pieces and soaked with 5% dilute ammonia by heating in 100 °C water for 24 hours followed by complete drying. Afterwards, the pieces were washed with ultrapure water and dried completely. Secondly, iron nitrate was dissolved in a solution of 50% ethanol and brought to a solution of 1.2 mol L^−1^ iron nitrate. Bamboo was fully soaked in 1.2 mol L^−1^ FeNO_3_, heated in 60 °C water for 5 d, and dried at 80 °C for 1 d. Third, the second step was repeated three times. After drying, the samples were heated to 600 °C and maintained in a muffle furnace for 3 h. After cooling, washing, and drying, the samples were used for subsequent experiments.

### 2.3. Laboratory-scale constructed wetland setup and operation

**Fig. 1:**
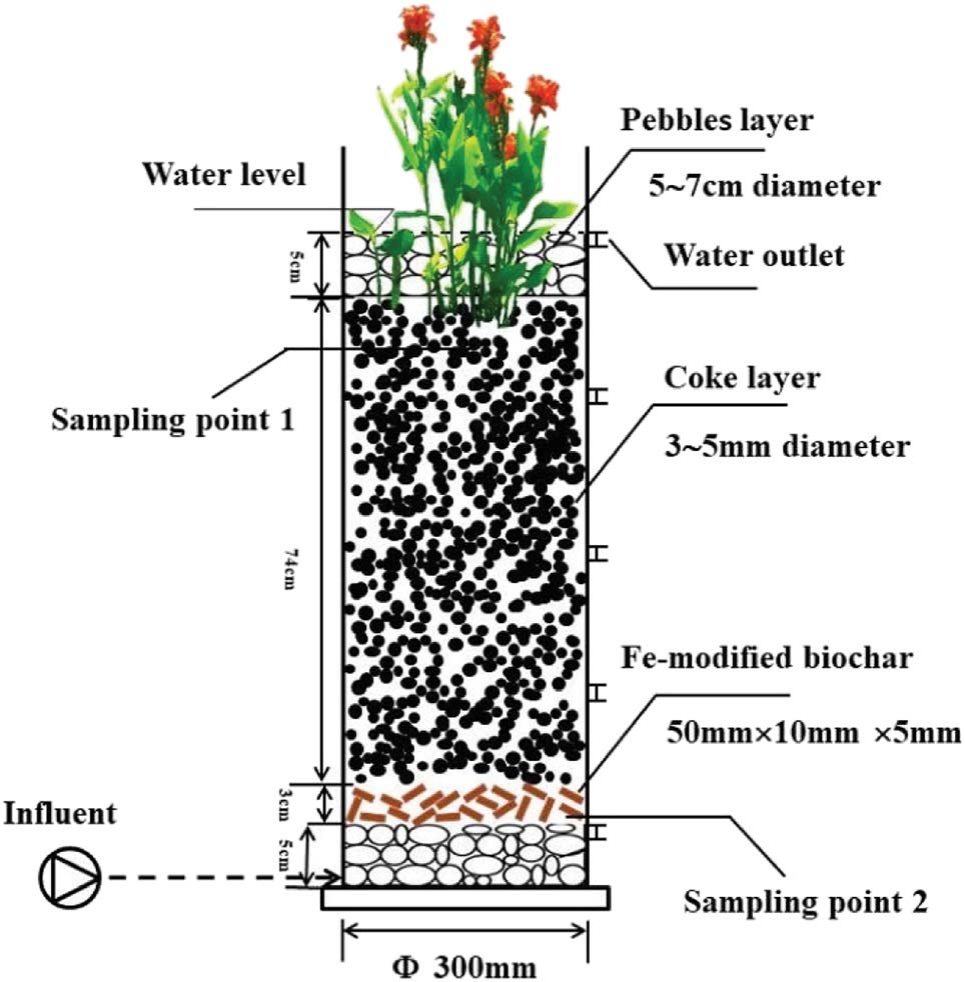
Schematic diagram of the CW3.

The CW units were placed in a greenhouse to avoid the impact of rainfall. Four UVCWs were constructed using PVC pipes that were 100 cm high and had an inner diameter of 5 mm (Fig. 1). The specific filling situation is shown in Table 1. The four CWs were divided into two groups consisting of two pairs. The units were named as described in Table 1.

**Table 1:** Filling situation of constructed wetland

Name high/cm	CW1	CW2	CW3	CW4
5	Pebbles	Pebbles	Pebbles	Pebbles
8	Quartz	Coke	Fe-modified biochar	Unmodified biochar
75	Quartz	Coke	Coke	Coke
80	Pebbles	Pebbles	Pebbles	Pebbles
85	Canna	Canna	Canna	Canna

### 2.4. Experimental procedure and sampling

**Fig. 2:**
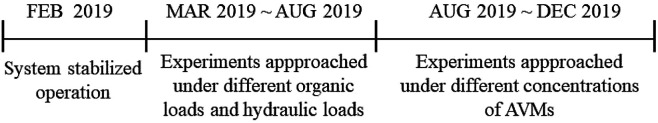
Division of the experimental period in the study.

This topical experiment was divided into two stages: the effect of the reactor on the removal of conventional pollutants under different load conditions and the removal of ABM of different concentrations in a reactor. The former provides some parameters for the latter. The experimental plan was divided into two parts (Fig. 2).

The experiment in the first stage was conducted from March 2019 to August 2019. The ability of each constructed wetland to remove COD, NH_4_^+^–N, and TP under different organic and hydraulic loads was tested to determine the optimal operating conditions of the CWs after homogenization of the wastewater taken from the effluent every 5 days for 4 months. The organic and hydraulic loads were established at three levels. The organic load was established as follows: low organic load, medium organic load, and high organic load. The hydraulic loads were as follows: low hydraulic load, medium hydraulic load, and high hydraulic load. Among them, three cycles were run under each organic load, and the hydraulic load was changed on this basis. Nine cycles were run for two one-week cycles. The combination of organic and hydraulic loads per cycle is shown in Table 2.

**Table 2:** Organic load and hydraulic load in each cycle

Period	Organic load	Hydraulic load
1	Low (62.50 mg L^−1^ ± 8.68%)	High (HLR = 5.66 m d^−1^)
2	Low (74.91 mg L^−1^ ± 5.76%)	Medium (HLR = 2.83 m d^−1^)
3	Low (65.88 mg L^−1^ ± 7.42%)	Low (HLR = 1.42 m d^−1^)
4	Medium (114.23 mg L^−1^ ± 7.66%)	High (HLR = 5.66 m d^−1^)
5	Medium (120.8 mg L^−1^ ± 9.90%)	Medium (HLR = 2.83 m d^−1^)
6	Medium (127.09 mg L^−1^ ± 6.65%)	Low (HLR = 1.42 m d^−1^)
7	High (229.52 mg L^−1^ ± 16.86%)	High (HLR = 5.66 m d^−1^)
8	High (241.11 mg L^−1^ ± 12.91%)	Medium (HLR = 2.83 m d^−1^)
9	High (225.30 mg L^−1^ ± 8.89%)	Low (HLR = 1.42 m d^−1^)

The second stage was prepared as follows: based on the optimal operating conditions of the CWs obtained in the first stage, it was applied to four CWs, and ABM was added to the simulated wastewater for the simulation of ABM wastewater. The experiment was operated from August 2019 to December 2019. The Fe-modified biochar was filled to the bottom of the device, and a peristaltic pump was used to continuously pump water from the bottom to top to study the ability of iron oxide to remove ABM in an oxygen-free environment. The ABM concentrations were established at three levels (Table 3). Each concentration was subjected to a cycle that was run for 30 d. After the experiment, the filler sample was sent to Sangon Biotech (Shanghai, China) for further analysis. The influent water was simulated polluted river water, and its characteristics are listed in Table S1.†

**Table 3:** ABM concentration in each cycle

Period	ABM concentration (mg L^−1^)
1	Low (0.106 ± 5.38%)
2	Medium (0.512 ± 5.18%)
3	High (1.056 ± 5.17%)

### 2.5. Sample extraction and clean up

Owing to the low content of ABM in wastewater that was extracted from the constructed wetlands, a solid-phase extraction method was used for pretreatment. The pretreatment methods were as follows: (1) water samples (400 mL) were filtered through a glass fiber membrane of 0.45 μm. (2) Oasis HLB extraction cartridges were activated with 5.0 mL of acetonitrile, followed by 5.0 mL of 40% of aqueous acetonitrile. (3) The filtered water sample of 300 mL was added and passed through the HLB solid-phase extraction column at a speed of 10 mL min^−1^ and then drained under vacuum for 30 min. (4) Acetonitrile/methanol = 3/2 (v/v) was used as the eluent; HLB was eluted with 5 mL eluent, and then the liquid was vacuumed and collected. (5) The eluent was dried with a stream of nitrogen, redissolved in 3 mL methanol, and then placed in a 2 mL brown sample bottle after filtration through a 0.22 μm membrane for HPLC analysis.

### 2.6. Instrument analysis

ABM was analyzed using an HPLC system of the Agilent 1260 series (Agilent Technologies, Santa Clara, CA, USA). The volume injected was 20 μL, and chromatography was performed at 40 °C. The mobile phase consisted of water (component A) : methanol (component B), 88 : 12 (v/v) that was pumped in at an initial flow rate of 1.0 mL min^−1^. Powder X-ray diffraction (XRD, X'Pert PRO, PANalytical, Almelo, Netherlands) was employed to analyze the chemical composition of Fe-modified biochar at the 80 cm layer. Scanning electron microscopy (SEM, JSM-7900V, JEOL, Tokyo, Japan) was used to observe the surface structure of Fe-modified biochar in CW3.

### 2.7. Substrate sampling and microbial abundance analysis

The microbes around the root of the plant and matrix filler were analyzed in more detail. The two samples, including the rhizosphere and biochar fillers, were collected in each UVCW (Fig. 1). All the samples were then frozen at −80 °C for transport. Finally, the samples were sent to Sangon Biotech for testing by metagenomic sequencing.

Pyrosequencing and Illumina high-throughput sequencing (Illumina, San Diego, CA, USA) were completed by Sangon Biotech. The specific methods were described in our previous study.^34^ Microbial DNA was amplified using a set of primers by targeting the V3–V4 hypervariable region of 16S rDNA. This is a common method to investigate the bacterial community composition. The sequence of forward primers was Nobar_341F (CCTACGGGNGGCWGCAG) and the reverse primers was Nobar_805R (GACTACHVGGGTATCTAATCC).

### 2.8. Statistical analysis

The COD, NH_4_^+^–N, and TP were determined using the latest Chinese standard methods.^35,36^ Principal component analysis (PCA) was used to compare and analyze the distribution of plant rhizosphere and underfill microbial communities. To evaluate the impact of different heights of constructed wetlands on the rate of removal of ABM, a one-way analysis of variance (ANOVA) was utilized.

## 3. Results

### 3.1. Effect of the removal of pollutants under different organic loads

**Fig. 3:**
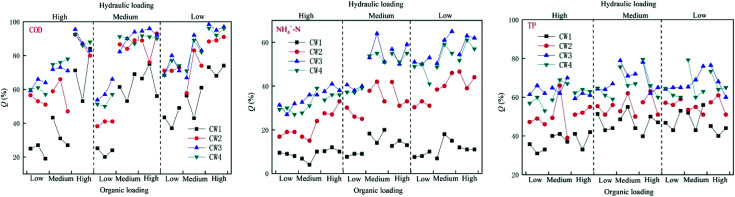
Effects of organic load on the removal of COD, NH_4_^+^–N and TP in constructed wetlands.

Under different organic loads, the four constructed wetlands had different treatment efficiencies for COD, NH_4_^+^–N, and TP. Under high organic loads, the average rate of removal of COD by CW1, CW2, CW3, and CW4 was 68%, 87%, 93% and 91%, respectively. The average rate of removal of NH_4_^+^–N was 11%, 35%, 51%, and 48%, respectively, and the average rate of removal of TP was 42%, 55%, 64%, and 64%, respectively. Under a medium organic load, the average rate of removal of COD by CW1, CW2, CW3, and CW4 was 49%, 74%, 82%, and 80%, respectively, and the average rate of removal of NH_4_^+^–N was 12%, 32%, 49% and 46%, respectively. The average rate of removal of TP was 46%, 53%, 65%, and 65%. Under a low organic load, the average rate of removal of COD by CW1, CW2, CW3, and CW4 was 29%, 55%, 65% and 61%, respectively. The average rate of removal of NH_4_^+^–N was 8%, 25%, 40%, and 37%, and the average rate of removal of TP was 42%, 52%, 65%, and 62%, respectively. Overall, the constructed wetlands with biochar more effectively treated conventional organic pollutants than the constructed wetlands without biochar. Because biochar strongly adsorbs organic pollutants in biochar constructed wetlands, the ability of biochar constructed wetlands to remove COD, NH_4_^+^–N and TP is generally higher than that of ordinarily constructed wetlands. CW3 is more effective at removing these compounds than CW4 (Fig. 3). In addition to the modified biochar being better able to adsorb pollutants, iron oxides are formed on the surface of the Fe-modified biochar, which greatly improves the absorption of nitrogen. Thus, the treatment of NH_4_^+^–N was highly notable.

**Fig. 4:**
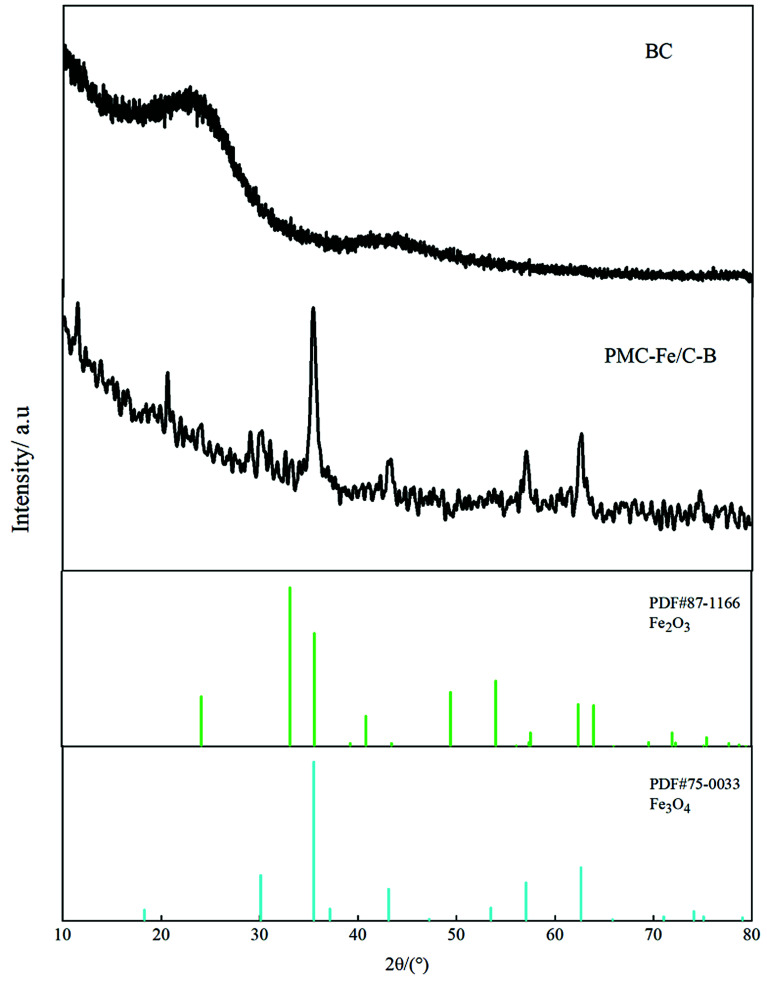
XRD patterns of Fe-modified biochar in CWs.

**Fig. 5:**
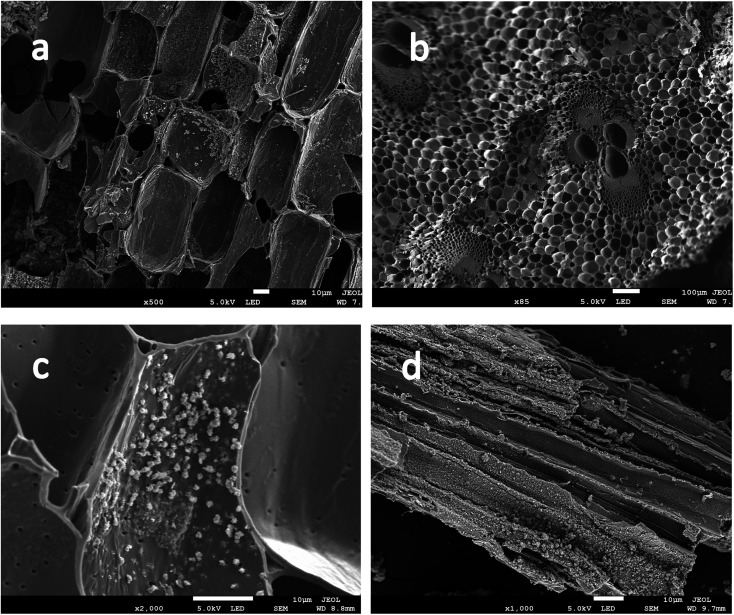
The SEM pictures of the Fe-modified biochar CW3.

### 3.2. Effect of the removal of pollutants under different hydraulic loads

**Fig. 6:**
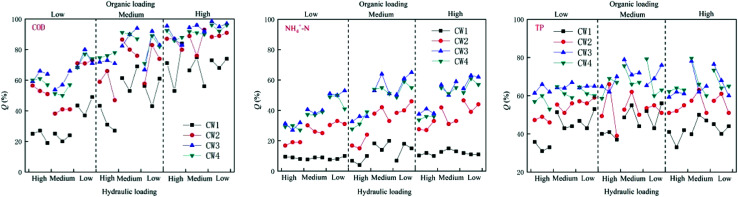
Effect of hydraulic load on the removal of COD, NH_4_^+^–N and TP in constructed wetlands.

Under different hydraulic loads, the four constructed wetlands had different treatment efficiencies for COD, NH_4_^+^–N, and TP (Fig. 6). Under high hydraulic loads, the average rate of removal of COD by CW1, CW2, CW3, and CW4 was 56%, 78%, 84%, and 83%, respectively; the average rate of removal of NH_4_^+^–N was 11%, 39%, 57% and 52%, respectively, and the average rate of removal of TP was 47%, 56%, 76%, and 74%. Under medium hydraulic loads, the average rate of removal of COD by CW1, CW2, CW3, and CW4 was 50%, 71%, 71%, and 78%, respectively, and the average rate of removal of NH_4_^+^–N was 13%, 33%, 50% and 48%, respectively. The average rate of removal of TP was 47%, 55%, 76% and 73%, respectively. Under low hydraulic loads, the average rate of removal of COD by CW1, CW2, CW3, and CW4 was 9%, 22%, 35%, and 32%, respectively, and the average rate of removal of NH_4_^+^–N was 13%, 33%, 50% and 48%, respectively. The average rate of removal of TP was 37%, 50%, 63% and 62%. By comparing the rate of removal of COD by the four artificial wetlands, it was apparent that the ability of the biochar constructed wetlands CW3 and CW4 to remove COD was better than that of the ordinary biochar constructed wetlands CW1 and CW2, which shows that biochar has a significant effect on the removal of COD in constructed wetland influences. Reducing the hydraulic load can increase the ability of the constructed wetlands to remove COD. The time of residence of sewage in the constructed wetlands was shorter at higher hydraulic loads, and the adsorption of organic pollutants by the substrates in the constructed wetlands was weaker than that of the water flow. The resistance of organic pollutants to flow, and at lower hydraulic loads, the resistance of the flow to organic pollutants decreases. The capacity of the matrix in the constructed wetland to adsorb organic pollutants is higher than the resistance to flow, so that the organic pollutants can be adsorbed in the matrix, thereby being subject to degradation by microorganisms as a carbon source.

### 3.3. Performance of different CWs at removing pollutants

**Fig. 7:**
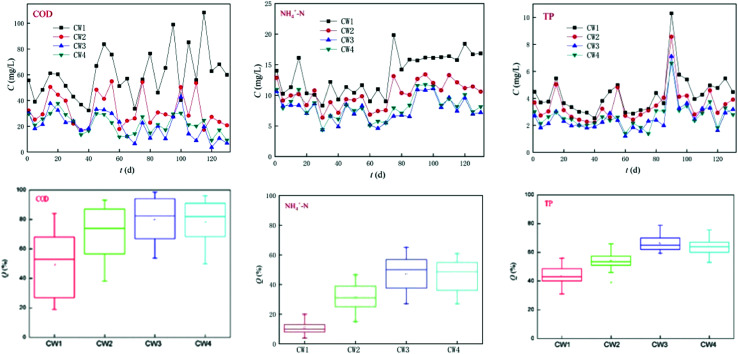
Removal effect of four constructed wetlands on conventional pollutants.

The four reactors ran smoothly within 120 days and effectively reduced the concentration of conventional pollutants (Fig. 7). CW3 is a constructed wetland supplemented with iron-modified biological bamboo charcoal, which is the most effective at removing conventional pollutants, and the highest removal rates of COD, ammonia nitrogen and TP can reach 98%, 65%, and 80%, respectively, while the average rate of removal was 80%, 50%, and 63% respectively. CW4 is a constructed wetland supplemented with unmodified biological bamboo charcoal (BC). Its rate of removal of conventional pollutants was slightly worse than that of CW3, and the highest rates of removal of COD, ammonia nitrogen, and TP can reach 96%, 61%, and 80%, respectively. The average rate of removal was 79%, 45%, and 61%, respectively. CW3 and CW4 can remove significant amounts of COD. The ability to remove TP is also good, but the removal of ammonia nitrogen was poor. CW2 and CW1 are ordinary coke and quartz sand constructed wetlands, both of which have a poorer efficiency of removal of COD than CW3 and CW4 in which the highest rate of removal of CW2 for COD, ammonia nitrogen, and TP was 88%, 47%, and 66%, respectively. The rate of removal was 70%, 30%, and 53%, respectively. The ability to remove COD and TP was better, but the ability to remove ammonia nitrogen was poor. The highest rate of removal of CW1 for COD, ammonia nitrogen, and TP was 84%, 20%, and 56%, and the average rate of removal was 56%, 10%, and 43%. The ability to remove COD and TP was poor, while the ability to remove ammonia nitrogen was extremely poor. A comparison of CW2, CW3, and CW4 indicates that the addition of biochar in artificial wetlands can significantly improve the removal of conventional pollutants.

### 3.4. Efficiencies of different CWs at removing ABM

**Fig. 8:**
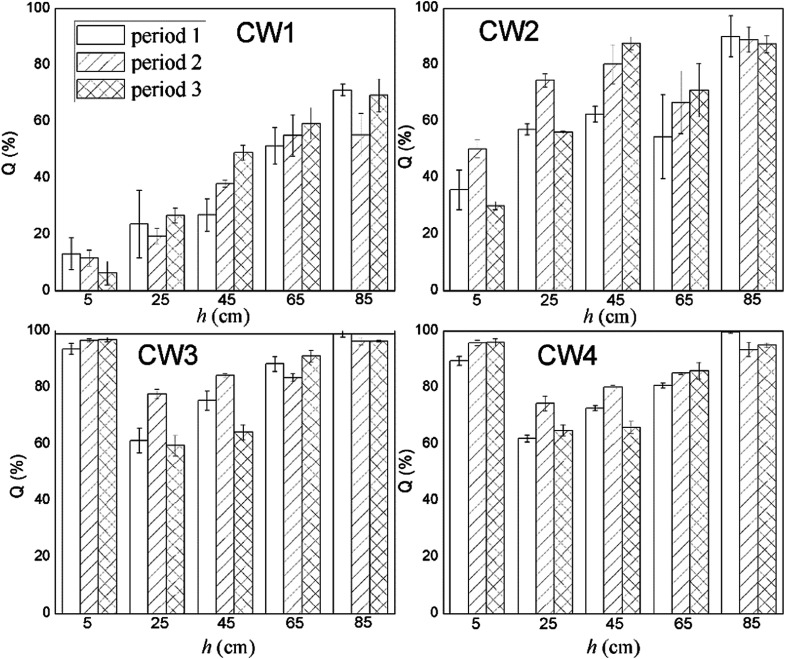
ABM removal rate along constructed wetlands.

**Fig. 9:**
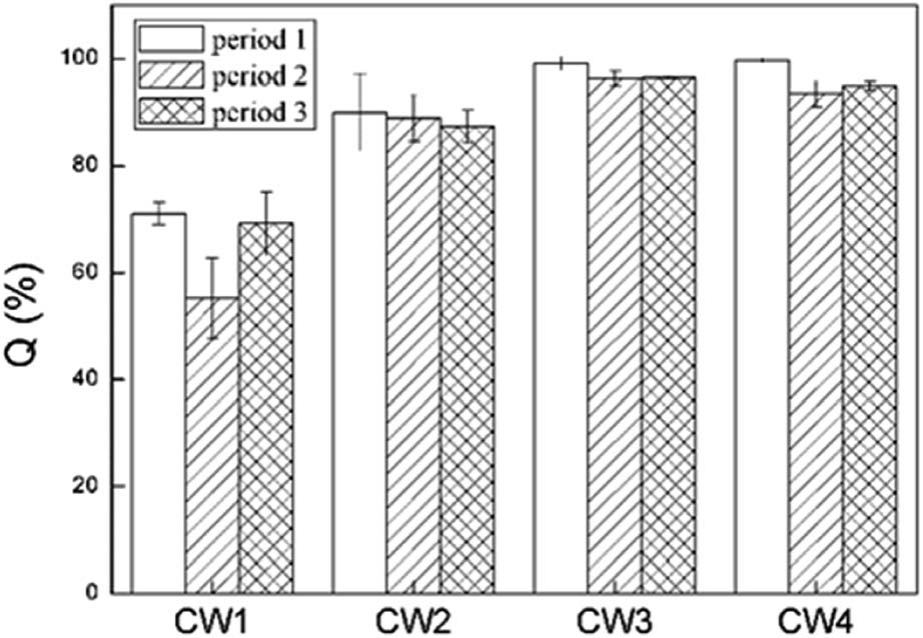
Removal effect of abamectin in four constructed wetlands.

Fig. 8 shows the rate of removal along with the concentration of ABM in a constructed wetland. In CW3, starting from a height of 5 cm, the cumulative efficiency at removing ABM improved significantly, particularly when compared with CW1 and CW2. In fact, in CW3, ABM was removed at a height of 5 cm, and the maximum cumulative removal efficiency was 96.0%. This is much higher than the top 15% of CW1. Comparing the ability to remove ABM at the 5 cm sampling port of the four constructed wetlands in the three cycles, the ability to purify ABM was CW3 > CW4 > CW2 > CW1 (Table S3†). The rate of removal of ABM by CW1 at 5 cm was less than 15%, while that of CW2 was between 30% and 50%. The highest efficiency of removal of ABM was at the height of 5 cm in CW3. This is also the height at which conventional pollutants were removed the most quickly.^37^ Moreover, the Fe-modified biochar exhibited a better adsorption capacity than the other matrix filler. Fig. 9 shows the total effectiveness of the four CWs at removing ABM. When the four CWs were operated under period 1, the treatment efficiency of CW1, CW2, CW3, and CW4 to ABM was 71.06% ± 2.05%, 90.05% ± 7.25%, 99.72% ± 1.25%, and 99.01% ± 0.42%, respectively. Under period 2, the treatment efficiency of the four CWs was 55.25% ± 7.62%, 88.95% ± 4.42%, 96.39% ± 1.38%, and 93.49% ± 2.47%, respectively. Under period 3, the treatment efficiency of the four CWs was 69.18% ± 5.91%, 87.33% ± 3.09%, 96.51% ± 0.26%, and 95.00% ± 0.81%, respectively. The ability of CW3 and CW4 to remove ABM was highly significant, and the rate of removal can reach more than 99%. The common constructed wetlands CW1 and CW2 were also somewhat able to remove ABM.

### 3.5. Shifts in microbial community structure

Different microbial environments affect the composition and quantity of the microbial community, thereby affecting the sewage removal effect.^1,38^ To investigate the composition of the microbial community, samples at sampling points 1 and 2 were selected from each UVCW for community structure analysis, and designated CW1 1, CW2 1, CW3 1, CW4 1, CW1 2, CW2 2, CW3 2, and CW4 2, respectively. The diversity indices revealed that the Shannon and Simpson values of the samples from the four UVCWs varied significantly.^39^ This indicates a shift of microbial community composition by the addition of Fe-modified biochar (Table S2†).

**Fig. 10:**
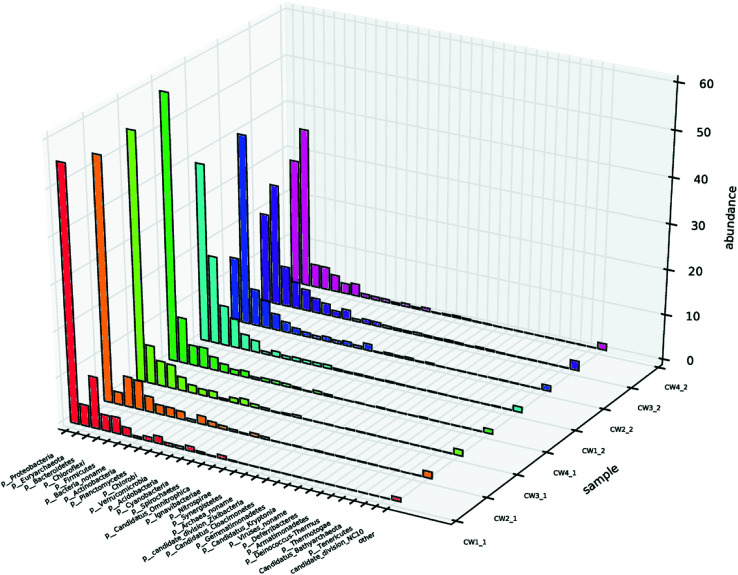
Microbial community structure on the surface and bottom layers of constructed wetlands.

The level of microbial community structure in the surface layer of constructed wetland after adding ABM wastewater is shown (Fig. 10). In terms of phyla, there were four that clearly stood out from the seven phyla that were identified in the surface layer of CW1: γ-*Proteobacteria*, β-*Proteobacteria*, δ-*Proteobacteria*, methane Microbacteria, α-*Proteobacteria*, anaerobic Orthomycetes, and *Bacteroides*. Fix carbon dioxide in the atmosphere to provide a carbon source for denitrification; *Proteobacteria* are also mostly denitrifying bacteria. β-*Proteobacteria*, γ-*Proteobacteria*, δ-*Proteobacteria*, Methanomicrobacteria, α-*Proteobacteria*, anaerobic streptococci, *Bacteroides*, and *Clostridia* were found in the bottom layers of CW2, CW3, and CW4 compared with CW1. Compared with CW1, β-*Proteobacteria* and δ-*Proteobacteria* in CW2 increased significantly, γ-*Proteobacteria* and α-*Proteobacteria* decreased significantly, and the rest of the fungi did not change significantly. δ-*Proteobacteria* in CW3 increased significantly, methane Micromycetes and β-*Proteobacteria* increased. γ-*Proteobacteria* and α-*Proteobacteria* decreased, while the rest of the fungi did not change significantly. δ-*Proteobacteria* increased significantly in CW4; β-*Proteobacteria* and methane Micromycetes increased, and the rest of the fungi did not change significantly. In this phylum, γ-*Proteobacteria*, and *Euryarchaeota* showed the greatest abundance in the substrate that was exposed to ABM.

### 3.6. Interactions between microbial communities and CW substrates

**Fig. 11:**
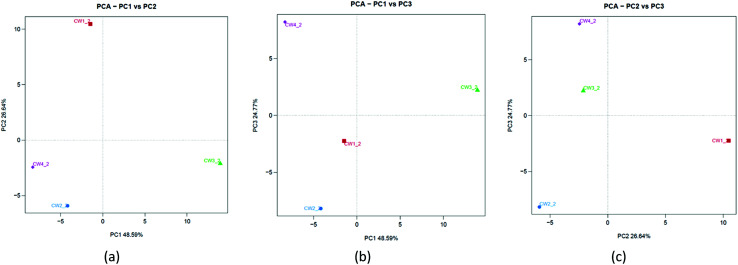
PCA analysis of the bottom microorganism community in constructed wetlands.

To understand the similarities between microorganisms and substrates of constructed wetlands, a principal component analysis was used in the study. Fig. 11a shows that PC1 and PC2 can explain 48.59% and 26.64% of the total variation, respectively. Fig. 11b shows that PC1 and PC3 can explain 48.59% and 24.77% of the total variation, respectively. Fig. 11c shows that PC2 and PC3 can explain 26.64% and 24.77% of the total variation, respectively. The three principal components were sufficient to explain approximately 100% of the total variance. This was different from the results of the PCA analysis at the physiological level. It showed that some microbial community structures were more sensitive to Fe-modified biochar than others.^40^ Although there were slight differences in the community composition and matrix-filler type, there was a common underlying community profile from a taxonomic perspective that revealed the dominance of a few phyla.^33^

## 4. Discussion

On the whole, the effect of four UVCWs at removing conventional pollutants (COD, NH_4_^+^–N, and TP) was CW3 > CW4 > CW2 > CW1. The addition of biochar in constructed wetlands can significantly improve the ability to remove conventional pollutants. The increase in organic load will increase the ability of a biochar constructed wetland to remove COD and NH_4_^+^–N. When the C/N ratio ranges from C : N = 2.5 : 1 to C : N = 5 : 1, the rate of removal of NH_4_^+^–N increases faster. When the C/N ratio ranges from C : N = 5 : 1 to C : N = 10 : 1, the rate of removal of NH_4_^+^–N increases slowly. The ability of a carbon constructed wetland to remove TP does not change with the change in organic load. A decrease in the hydraulic load will increase the ability of a biochar constructed wetland to remove COD and NH_4_^+^–N. With the decrease in the hydraulic load, the ability of CW3 and CW4 to remove COD and NH_4_^+^–N increased. It is hypothesized that the reason may be that the reduction of hydraulic load increases the time that pollutants reside in the biochar matrix, which improves the effect of adsorption and denitrification. The ability of a biochar constructed wetland to remove TP does not change with the change in hydraulic load. It can also quickly reach maximum adsorption of TP under these conditions. Even under a poor hydraulic load, this type of wetland can quickly adsorb the maximum amounts of TP. This could be because the removal of phosphorus primarily relies on adsorption and biochemical reactions, and biochar can accelerate the adsorption of phosphorus. The experimental results also show that biochar is a good filler for constructed wetlands. This is because the surface of biochar is rich in organic functional groups and has a stronger electron exchange.^41^ Biochar can accelerate the degradation of organic matter. The biochar modified by FeNO_3_ has a more positive charge, which could have increased its ability to adsorb NO_3_^−^–N and facilitate bacterial adhesion.^4,42^ Even if the adsorption capacity of biochar is exhausted, the biofilm formed on the biochar will continue to remove organic matter.^42^

SEM shows the surface morphology of Fe-modified biochar in Fig. 5b. The surface of Fe-modified biochar shows that the iron oxide has been successfully loaded and provides more adsorption sites to adsorb pollutants (Fig. 5a and c).^43,44^ As can be seen in Fig. 5d, the surface of Fe-modified biochar is rough, and the tubular pore structure can be easily observed. XRD was used to study the structure and phase purity of Fe-modified biochar composites. The main crystal phases of the Fe-modified biochar are carbon, quartz, γ-Fe_2_O_3_, and Fe_3_O_4_ (Fig. 4). The transfers of electrons between Fe(ii) and Fe(iii) play a role in an enormous range of environmental processes from mineral formation and dissolution to contaminant remediation.^31^ It has been previously reported as the main driver of abiotic Fe(ii) oxidation during nitrate reduction.^45^ It was also proven that Fe^2+^ promoted the denitrification process when there was a lack of organic matter in the treatment.^29^ The process of oxidation of iron coupled to the reduction of nitrate is as follows:^43,45^

1 10Fe^2+^ + 24H_2_O + 2NO_3_^−^ → 10Fe(OH)_3_ + 18H^+^ + N_2_

There is no significant difference in microbial species and abundance in sampling point 1 of the four CWs. However, in sampling point 2, the microbial abundance of CW3 was lower than that of the other CWs. One can hypothesize that the reason may that the Fe-modified biochar modified by iron is only suitable for the growth of specific microorganisms.^46^ This may provide direct evidence for the evolution of the microbial community structure in iron-driven wastewater treatment.^29^

The presence of ABM leads to changes in the relative abundance of bacterial populations in the CW substrates.^36^ Antibiotics are specifically designed for bacteria, and different concentrations of antibiotic wastewater interfere with the function of bacterial char conversion. Simultaneously, bacterial communities with drug resistance can adapt to this environment but still retain the characteristics of functional diversity.^38^ Previous studies showed that the constructed wetlands with Fe-modified biochar can effectively improve the efficiency of nitrogen removal, but in this study, there was no difference in the efficiency of nitrogen removal between CW3 and CW4 (*P* > 0.05). This was probably owing to the fact that the amount of Fe-modified biochar was too small, and ability to remove nitrogen was not high. Alternatively, synthetic simulated wastewater cannot be treated as real agricultural wastewater. The nutrient concentrations in simulated wastewater were low, and the systems were not limited by oxygen availability.^14^

In previous studies, El-Khateeb *et al.*^23,24,47^ combined constructed wetlands with wastewater treatment technology to achieve wastewater treatment efficiency and improve effluent quality. However, from the perspective of the treatment efficiency of constructed wetlands, that of ordinary constructed wetlands is lower than that of constructed wetlands that were modified with Fe. If Fe-modified constructed wetlands are used as terminal water treatment facilities and combined with other water treatment technologies, water treatment efficiency could be improved and reduce losses of energy.

## 5. Conclusions

Constructed wetlands are a promising technology to treat agricultural wastewater. However, the lack of detailed design and operational data makes them difficult to promote. In this study, we adjusted the different hydraulic load and organic load in the early stage to determine the operating conditions for the best treatment efficiency of the UVCWs. The enhanced removal of ABM and other pollutants in the CWs was obtained *via* an improvement in the adsorption capacity of the substrate and a higher abundance of microorganisms for biodegradation using Fe-modified biochar. The Fe-modified biochar provided electron acceptors to improve the microbial degradation of ABM in the substrate. Under hypoxia and aerobic conditions, not only the microbial structure changes in the Fe cycle but also the delta *Proteobacteria* and Methanomicrobia are abundant, which ultimately improves the ability of CW3 to remove ABM. In summary, in anoxic and aerobic areas, the circulation of iron in chemical waste between Fe(ii) and biogenic Fe oxides does indeed establish and enhance the removal of pollutants. Therefore, one of the future research topics will examine how to degrade organic compounds through electron transfer between microorganisms. Fe-modified biochar is a potential carrier of microbial agents. The interaction between biochar, microorganisms, and plants can provide a green method to repair polluted water bodies. In nature, we will further discuss the mechanism of biochar-plant-microorganisms and the impact on wastewater treatment in constructed wetlands. How to solve the potential risk of the accelerated accumulation of antibiotic resistance genes in CW is also a problem that requires urgent action.

## Conflicts of interest

The authors declare no conflicts of interest.

## Supplementary Material

RA-010-D0RA08265A-s001
