# Supplementary material for: Removal of abamectin and conventional pollutants in vertical flow constructed wetlands with Fe-modified biochar
Source: RSC Adv. 2020 Dec 15;10(72):44171–82. doi: 10.1039/d0ra08265a (PMC9058508; doi:10.1039/d0ra08265a)
Supplement: RA-010-D0RA08265A-s001 [file RA-010-D0RA08265A-s001.pdf]

Electronic Supplementary Material (ESI) for RSC Advances.
This journal is © The Royal Society of Chemistry 2020

Table S1 Characteristic of the influence water

	NH_4-N	COD	TP	pH
Concentration（mg/L）	10-20	50-200	5-10	7.51 ± 0.15

Table S2 Statistical table of genetic diversity

Height/cm	Sample	OTUs	Shannon	Simpson
80	CW1-80	1374204	12.4097	0.000023
	CW2-80	1412532	13.017767	0.000008
	CW3-80	1695305	12.820155	0.000019
	CW4-80	1368255	11.948025	0.000064
20	CW1-20	1491461	13.034722	0.000007
	CW2-20	1209483	12.306531	0.000021
	CW3-20	1335816	12.889573	0.000009
	CW4-20	1213775	12.530142	0.000015

(Samples with heights of 20-30 cm and 70-80 cm were selected from four constructed wetlands which were named CW1-80, CW2-80, CW3-80, CW4- 80, CW1-20, CW2-20, CW3-20, CW4-20.)

Table S3 Removal rate of abamectin in each sampling mouth of four simulated constructed wetlands

Name	High（cm）	Removal rate（%）	Name	High（cm）	Removal rate（%）	Name	High（cm）	Removal rate（%）	Name	High（cm）	Removal rate（%）
CW-1 Period 1	5	13.06%±5.56%	CW-2 Period 1	5	35.73%±6.98%	CW-3 Period 1	5	93.62%±1.99%	CW-4 Period 1	5	89.42%±1.67%
	25	23.61%±11.94%		25	57.15%±1.97%		25	61.15%±4.34%		25	61.98%±1.29%
	45	26.86%±5.75%		45	62.53 %±2.84%		45	75.43 %±3.34%		45	72.66 %±0.81%
	65	51.35%±6.53%		65	54.58%±14.92%		65	88.32%±2.61%		65	80.73%±0.85%
	85	71.06%±2.05%		85	90.05%±7.25%		85	99.72%±1.25%		85	99.01%±0.42%
CW-1 Period 2	5	11.58%±2.83%	CW-2 Period 2	5	50.3%±3.06%	CW-3 Period 2	5	96.72%±0.57%	CW-4 Period 2	5	95.56%±1.31%
	25	19.38%±2.65%		25	74.52%±2.39%		25	77.76%±1.71%		25	74.39%±2.68%
	45	37.91%±1.18%		45	80.0.%±6.88%		45	84.31%±0.61%		45	80.12%±0.60%
	65	55.04%±7.33%		65	66.73%±11.05%		65	83.50%±1.41%		65	85.13%±0.48%
	85	55.25%±7.62%		85	88.95%±4.42%		85	96.39%±1.38%		85	93.49%±2.47%
CW-1 Period 3	5	6.29%±4.15%	CW-2 Period 3	5	30.14%±1.43%	CW-3 Period 3	5	96.98%±0.75%	CW-4 Period 3	5	95.79%±0.96%
	25	26.66%±2.65%		25	56.19%±0.31%		25	59.52%±3.61%		25	64.88%±1.93%
	45	48.86%±2.67%		45	87.65%±2.20%		45	64.17%±2.62%		45	65.97%±2.10%
	65	59.25%±5.66%		65	71.02%±9.43%		65	91.13%±2.13%		65	85.99%±2.92%
	85	69.18%±5.91%		85	87.33%±3.09%		85	96.51%±0.26%		85	95.00%±0.81%
